# Efficacy of phage cocktail AB-SA01 therapy in diabetic mouse wound infections caused by multidrug-resistant *Staphylococcus aureus*

**DOI:** 10.1186/s12866-020-01891-8

**Published:** 2020-07-09

**Authors:** Legesse Garedew Kifelew, Morgyn S. Warner, Sandra Morales, Lewis Vaughan, Richard Woodman, Robert Fitridge, James G. Mitchell, Peter Speck

**Affiliations:** 1 College of Science and Engineering, Flinders University, Adelaide, South Australia Australia; 2 St Paul’s Hospital Millennium Medical College, Addis Ababa, Ethiopia; 3 Infectious Diseases Unit, The Queen Elizabeth Hospital, Woodville, South Australia Australia; 4 Faculty of Medicine, University of Adelaide, Adelaide, South Australia Australia; 5 AmpliPhi Biosciences Corporation, Sydney, New South Wales Australia; 6 Research Development and Support, Flinders University, Adelaide, South Australia Australia; 7 College of Medicine and Public Health, Flinders University, Adelaide, South Australia Australia; 8 Discipline of Surgery, The University of Adelaide, Adelaide, South Australia Australia

**Keywords:** Diabetic mice, Infection, MDR *S. aureus*, Phage cocktail, Treatment, Wound

## Abstract

**Background:**

Diabetic foot ulcer (DFU) is a serious complication of diabetes mellitus. Antibiotic-resistant *Staphylococcus aureus* is frequently isolated from DFU infections. Bacteriophages (phages) represent an alternative or adjunct treatment to antibiotic therapy. Here we describe the efficacy of AB-SA01, a cocktail of three *S. aureus Myoviridae* phages, made to current good manufacturing practice (cGMP) standards, and which has undergone two phase I clinical trials, in treatment of multidrug-resistant (MDR) *S. aureus* infections.

**Results:**

Wounds of saline-treated mice showed no healing, but expanded and became inflamed, ulcerated, and suppurating. In contrast, AB-SA01 treatment decreased the bacterial load with efficacy similar or superior to vancomycin treatment. At the end of the treatment period, there was a significant decrease (p < 0.001) in bacterial load and wound size in infected phage- and vancomycin-treated groups compared with infected saline-treated mice. In phage-treated mice, wound healing was seen similar to vancomycin treatment. No mortality was recorded associated with infections, and post-mortem examinations did not show any evident pathological lesions other than the skin wounds. No adverse effects related to the application of phages were observed.

**Conclusion:**

Topical application of phage cocktail AB-SA01 is effective, as shown by bacterial load reduction and wound closure, in the treatment of diabetic wound infections caused by MDR *S. aureus*. Our results suggest that topical phage cocktail treatment may be effective in treating antibiotic-resistant *S. aureus* DFU infections.

## Background

Complications of diabetes, such as diabetic foot ulcers (DFUs), are common, multifactorial in origin, and costly [1]. The global burden of DFUs is rising, affecting up to 26.1 million people each year [2]. DFUs are the precipitating cause for nearly 90% of limb amputations among persons with diabetes [3]. Worldwide, 6.3% of persons living with diabetes are affected by DFUs, and the lifetime incidence of a foot ulcer among persons with diabetes is estimated between 19 and 34% [2]. The 5-year mortality rate following foot amputation due to DFUs has been estimated at up to 74% [4]. DFU management is costly because it may involve imaging studies, revascularization, wound dressing for lengthy periods, debridement, antibiotic therapy, and management of metabolic abnormalities [5, 6]. In the USA, the annual cost of DFU management is estimated at an additional $9–13 billion over the cost of diabetes itself [7]. In England, it is estimated that the annual cost of managing DFUs exceeds the total cost of breast, prostate, and lung cancers combined [8].

*S. aureus* is a virulent pathogen frequently isolated from DFU [5, 9, 10]. A study in Australia found that two-thirds of DFU patients were infected with *S. aureus*, and nearly half were methicillin-resistant (MRSA), for which there are limited antimicrobial treatment options [11]. Hence, antibiotic resistance is a major obstacle in treating infections caused by this pathogen [5, 12]. Bacteriophages (phages, viruses that infect bacteria) represent an alternative or adjunct therapy to antibiotics. Lytic phages kill their bacterial host by lysis (bursting the infected bacterial cell to release progeny phages) [13]. Before the advent of antibiotics in the 1940s, phages were widely used in the USA and Europe. However, following the success of antibiotics, phage treatment was excluded from Western medicine, while it has continued to be practiced in Eastern Europe for > 100 years to treat bacterial infections [14]. The process of phage infection and subsequent self-replication in bacteria offers advantages over antibiotics: phages amplify themselves at the infection site provided there are sensitive bacterial hosts [15]. Phages are specific for the bacterial species they infect, an advantage over broadly active antimicrobials, as phages are not expected to disrupt a patients’ normal microflora [16, 17]. Phages lyse biofilm structures of bacteria, such as those typically found in infected DFUs [15, 18].

There is much evidence that phage use is safe (reviews: [19, 20]), and its extensive Russian and Georgian use has few adverse event reports [19]. Phages only infect bacteria, and are the most common biological entities in the biosphere and human body [21]. In 2006, the US Food and Drug Administration (FDA) gave “Generally Regarded As Safe” (GRAS) status to phage product Listex, against *Listeria monocytogenes* so that it can be added to human foods as a processing aid [22]; many phage products in this industry now have such status.

The physician from Phagebiotics Research Foundation used Eliava Institute, Republic of Georgia, *S. aureus* phage Sb-1, to successfully treat five patients with DFU infections that were unresponsive to standard antibiotics and which left the patients facing potential amputations. In each case, full wound closure was achieved, and the toes were saved [23]. Further studies with a product that meets cGMP regulations would support the uptake of this treatment to broader Western medicine. We used a cocktail of three phages that are related to *Staphylococcus* phage K, designated AB-SA01 [24], which has undergone two phase I clinical trials and is made to cGMP standard. A previous study confirmed that AB-SA01 showed no or minimum off-target effects [24]. The objective of this work was to determine the efficacy of this cGMP phage product in treating *S. aureus* infection in a mouse model in which diabetes is induced by administration of the naturally occurring antineoplastic agent streptozotocin. Here we report the efficacy of this phage preparation in treating MDR (resistant to benzylpenicillin, oxacillin, ciprofloxacin, erythromycin, and clindamycin) *S. aureus* infected deep skin wounds in diabetic mice.

## Results

### Confirmation of diabetic mouse infection model

Female Balb/c mice rendered diabetic by intraperitoneal (IP) injection of streptozotocin (STZ) were used in this study. Most of the diabetic mice manifested sustained hyperglycaemia and continued weight loss in the first 2 weeks post-STZ injection. Subjective observation of cage litter showed increased urine production requiring daily rather than every 3rd day litter changes, consistent with polyuria. Additionally, water intake by diabetic mice was estimated to be double their pre-STZ injection intake, indicative of polydipsia.

Forty (83.3%) mice developed diabetes with non-fasting blood glucose level (BGL) ≥ 13.9 mmol/L within the first 2 weeks of post-STZ administration. Of the remaining mice, 3 (6.3%) failed to develop diabetes, and 5 (10.4%) died before their diabetes status was determined. Of the diabetic mice, 21 (52.5%) developed severe hyperglycaemia. While the mean body weight of mice before STZ administration was 18.9 ± 0 g, the mean body weight of normal glycaemic, moderately hyperglycaemic, and severely hyperglycaemic mice groups were 19.3 ± 1.0, 17.8 ± 1.4, and 16.7 ± 1.4 g, respectively, at the end of the diabetes maintenance period. Neither body weight gain nor BGL decrease differed significantly between moderately and severely hyperglycaemic groups in the insulin treatment period (p > 0. 05).

Prior to the commencement of the wound infection treatment study, mice that failed to maintain body weight were euthanized (n = 7). Diabetic mice then were inoculated with 6.7 log_10_ (CFU) MDR *S. aureus* SA63–2498 or 50 μl of saline directly to the bilateral splinted excisional wounds on the dorsum, overlaid with gauze, and covered with Opsite. MDR *S. aureus* SA63–2498 was susceptible to vancomycin and phage cocktail AB-SA01 and its components. Gauze patches (10 × 10 mm) soaked with 70 μl AB-SA01, equivalent to 7.9 log10 PFU, or 70 μl saline solutions for the control group were applied every other day for 3 days starting from the day 3 of infection. Vancomycin-treated mice received 150 mg/kg vancomycin IP twice daily for five consecutive days. No mortality was recorded associated with infections, and post-mortem examinations did not show any evident pathological lesions other than the skin wounds.

### Efficacy of AB-SA01 on bacterial load in diabetic mouse wound infections

**Fig. 1:**
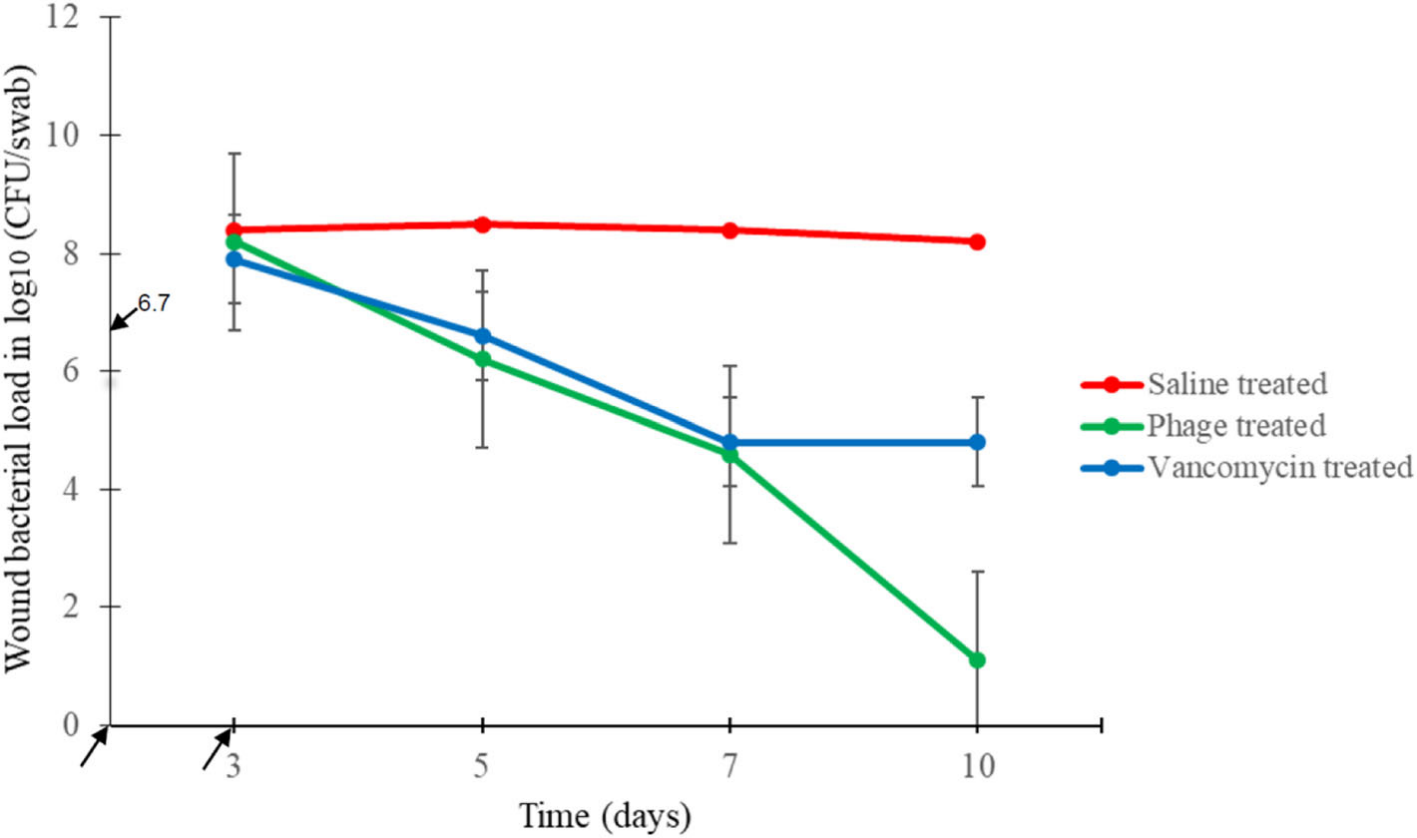
Effect of phage treatment on *S. aureus* bacterial load compared to control and IP vancomycin treatment. Saline-treated mice (control, red); AB-SA01 phage-treated mice (green); vancomycin-treated mice (blue). No *S. aureus* was detected from uninfected phage-treated mice. The arrows on the horizontal axis indicate the infection date (first arrow, day 0) and treatment start date (second arrow, day 3), respectively. Treatments were applied from day 3 to day 7 of infection, as detailed in the text. The arrow on the vertical axis indicates the infection dose

All infected wounds (no = 42) of the 21 mice yielded *S. aureus,* MDR SA63–2498, and the mean bacterial cell count ranged from 7.0–9.0 log_10_ (CFU/swab) with median 8.1 log_10_ (CFU/swab). The mean bacterial cell count on day 3 of infection from swab samples was 8.1 log_10_ (CFU/swab). The bacteria count on days 5, 7 and 10 of infection showed a significant decrease in all phage- and vancomycin-treated mice, as shown in Fig. 1.

No *S. aureus* was detected from more than half (*n* = 5/8) of phage-treated wounds, and the mean bacterial load from this group was 1.1 log_10_ (CFU/swab) on day 10 of the infection period with the maximum detection 4.3 log_10_ (CFU/swab). In contrast, minimum 3.9 log_10_ (CFU/swab) and mean 4.8 log_10_ (CFU/swab) *S. aureus* cells were detected in vancomycin-treated mice on day 7 and at the end of the experiment, respectively, as shown in Table 1.

**Table 1:** Bacterial load (log_10_ CFU/swab) after treatments in diabetic mice wounds infected with MDR *S. aureus*

Mouse ID	Treatment group	Infection period				
		Day 0	Day 3	Day 5	Day 7	Day 10
4-NEM	Saline	0	8.99	8.36	8.51	8.57
4-1 L		0	7.72	8.11	8.43	7.91
4-2R		0	8.34	8.85	8.38	8.18
5-NEM		0	7.00	8.34	8.41	7.62
5-1 L		0	8.76	8.70	8.20	8.11
5-2R		0	6.97	7.73	8.51	8.53
10-RL		0	7.00	8.38	8.26	7.95
Mean + err		0	8.42 + 0.9	8.47 + 0.4	8.39 + 0.1	8.23 + 0.3
7-1R	Phage	0	8.40	6.43	2.26	0.00
7-1 L		0	8.40	6.18	5.72	2.00
7-RL		0	7.88	6.45	6.30	2.28
7-2R		0	8.56	6.26	6.63	0.00
9-NEM		1.1	8.38	6.28	2.48	4.34
9-1R		1.5	8.81	6.45	5.15	0.00
9-1 L		0	8.41	5.80	5.72	0.00
9-RL		0	6.66	5.43	2.32	0.00
Mean + err		0.3	8.19 + 0.7	6.16 + 0.4	4.57 + 1.9	1.08 + 1.6
6-1R	Vancomycin	0	8.64	6.18	5.15	7.04
6-RL		0	7.46	4.70	3.96	5.86
6-2R		0	8.04	7.99	4.75	6.75
8-NEM		0	7.84	6.49	5.58	1.04
8-1 L		0	7.80	6.52	4.28	6.04
8-2R		0	7.72	7.92	4.96	2.11
Mean + err		0	7.92 + 0.4	6.63 + 1.2	4.78 + 0.6	4.81 + 2.6

A statistically significant (*p* < 0.001) bacterial load decrease was observed on day 5 of infection for the infected phage-treated group. The vancomycin-treated mice showed a statistically significant (*p* < 0.05) bacterial load reduction on day 5, and a more pronounced reduction (*p* < 0.001) was observed on day 7 of infection. On day 10 of infection, the bacterial load reduction due to phage cocktail treatment was statistically significantly (*p* < 0.05) less than vancomycin treatment. There was no *S. aureus* detected from uninfected phage-treated mice wounds throughout the experimental period.

### Wound healing

**Fig. 2:**
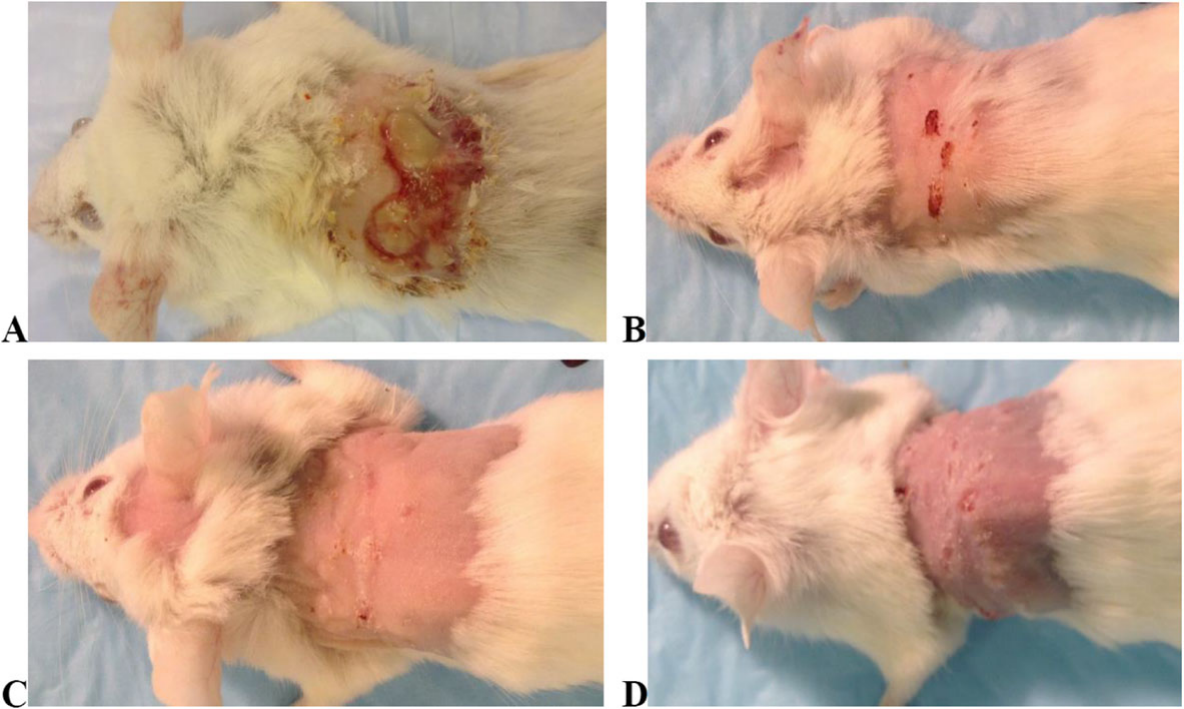
Representative images diabetic mouse wound at day 10 of infection: **A** infected saline-treated, showing lack of healing and expansion of wounds; **B** uninfected phage-treated, **C** infected phage-treated, and **D** infected vancomycin-treated. Wounds in **B, C,** and **D** groups showed similar complete healing

Infected phage- and vancomycin-treated mice showed a decrease in wound size during and after the course of treatment, leading to complete wound healing. All uninfected phage-treated wounds also decreased in size and had complete healing. In contrast, infected saline-treated wounds increased in size, as shown in Fig. 2.

**Fig. 3:**
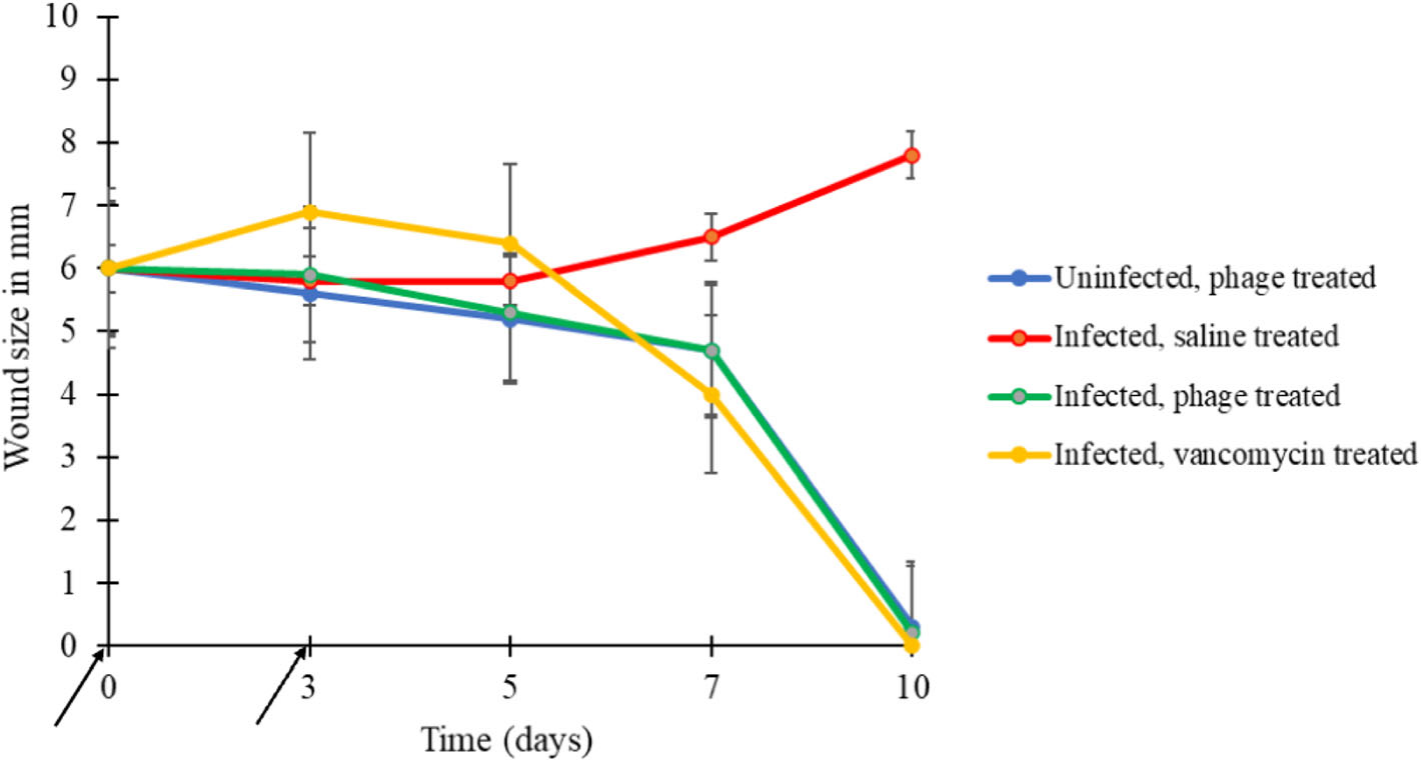
Effect of phage AB-SA01 on wound healing. The arrows on the horizontal axis indicate the infection date (first arrow, day 0) and treatment started date (second arrow, day 3). No healing was seen in infected saline-treated mice. In contrast, complete wound healing was seen in uninfected phage-treated wounds, and infected wounds treated with phage, and in the control group with infected wounds and vancomycin treatment

The mean wound diameter at the end of the experiment was 0.0, 0.2 (0.0–1.5), 0.3 (0.0–1.3), and 7.8 (6.1–9.0) mm for infected vancomycin-, infected phage-, uninfected phage-, and infected saline-treated groups, respectively. All infected saline-treated wounds manifested non-healing ulcers characterised by purulent exudate, discoloured granulation tissue, and foul odour. Compared to the infected saline-treated mice wound size, we found a significant (*p* < 0.05) wound size decreased on days 5, 7, and 10 of infection for infected phage-treated mice, as illustrated in Fig. 3.

There was a significant decrease (*p* < 0.001) in wound size in uninfected phage-, and infected phage-, and infected vancomycin-treated groups compared with infected saline-treated mice. There was no statistically significant difference (*p* > 0.05) in wound healing progression among uninfected phage-, infected phage-, and infected vancomycin-treated groups. Gross examination of the healed wounds showed no differences between these three treatment groups. Phage treatment caused no apparent adverse effects in mice in the absence or presence of its bacterial host.

### Clinical and postmortem assessment

Hunching, ruffled coat, lethargy, cold to touch, crinkling of skin, sunken eyes, and rapid or labored breathing were criteria used to check the health of mice. No mice manifested clinical signs of systemic infection during the infection and treatment periods. Non-healing wounds with purulent exudate, discolored granulation tissue, and foul odor were noted in all infected mice before treatment for all treatment groups, and the entire period of the experiment in the untreated groups. No gross pathological lesion was observed in any of the visceral organs examined during post-mortem investigation.

## Discussion

An inbred, chemically induced diabetic mouse (Balb/c) model was employed to assess the efficacy of AB-SA01 to treat MDR *S. aureus* infection in diabetic mice wounds. Multiple low doses of STZ resulted in a high proportion of diabetic mice, as reported [25, 26]. Skin wound healing in rodents involves skin contraction, a healing mechanism not seen in human wound healing, which occurs by secondary intention, granulation, and re-epithelialization [27, 28]. We employed a splint wound model to better mimic human skin wounds by avoiding the contraction type of mice wound healing, as described recently [27, 29]. We found that the silicone splints remained fixed on the mice’s skin until the end of the experiment.

Treatment of staphylococcal diabetic foot infections with antibiotics is becoming increasingly difficult because of the widespread presence of antibiotic-resistant *S. aureus* strains [10, 11, 30]. Previous reports have shown the potential of phages as an alternative or adjunctive therapy to treating bacterial infections in an animal model [31, 32], but information regarding the treatment of human DFUs caused by MDR *S. aureus* using a phage cocktail topically is limited. In this study, the therapeutic potential of AB-SA01 was tested on diabetic mice to treat wounds infected with an *S. aureus* clinical isolate resistant to multiple antibiotics, including those most commonly used to treat staphylococcal infections such as oxacillin, clindamycin, and trimethoprim-sulfamethoxazole. Inflicted wounds were infected the same day that the wounding occurred. After a week of treatment, AB-SA01 reduced bacterial loads to minimal levels while the control mice were still infected with a high bacterial load. A statistically significant (*p* < 0.001) viable bacterial load decrease was observed on day 5 and it continued to decrease throughout the infection period in AB-SA01-treated mice. The infection process, in terms of bacterial load and wound size, in phage- and vancomycin-treated groups was controlled well (*p* < 0.001) compared to untreated control mice.

The bacterial load in the vancomycin-treated group continued to decrease at a similar trajectory to the phage-treated group during the treatment period but remained constant from the day treatment was completed. The bacterial load reduction at the end of the experiment in vancomycin-treated mice was not as great and was significantly different (*p* < 0.05) from that in phage-treated mice. The continued bacterial load reduction in phage-treated mice after the treatment was stopped demonstrates an advantage of phages over antibiotic treatment, because phages replicate in the presence of their bacterial host [33, 34]. Of the eight infected phage-treated mice, *S. aureus* had been eradicated from the wounds of five mice by the end of the experiment. In the three remaining mice with detectable *S. aureus*, bacterial load was minimal compared to that in saline-treated mice. In contrast to phage-treated mice, at the end of the experiment, *S. aureus* was still detected from all the vancomycin-treated mice with mean bacterial load of 4.81 log_10_ (CFU/swab). Four of the six vancomycin-treated mice had a residual bacterial burden of ≥5.86 log_10_ (CFU/swab) at the end of the experiment.

From these observations, we conclude that AB-SA01 treatment could result in superior or equivalent efficacy to vancomycin, the usual first-line antimicrobial used to treat severe methicillin-resistant *S. aureus* infections. The data suggest that phages may be useful as an alternative therapy to antibiotics in this setting because they: a) may be used topically, b) show promising preliminary efficacy with a small number of treatments, shown here with three topical administrations, which is more compatible with standard wound care, c) show no local inflammatory reaction, d) may continue to work for an extended period post-administration, and e) have fewer documented adverse effects as compared to antibiotics such as vancomycin [35, 36]. Future controlled experiments are needed to determine the synergy of phage and antibiotic treatment.

Some reports suggest the effects of topical application of phages might be enhanced by simultaneous appropriate wound debridement [32, 37]. The effectiveness of this phage treatment that employed gentle debridement, topical application of a phage cocktail, and covering with light dressings such as gauze and Opsite, may represent a method for developing better diabetic wound care.

The use of well-developed phage cocktails (instead of single phage) not only broadens the spectrum of activity of therapeutic phage formulations but also reduces the likelihood of development of phage resistant bacteria [38, 39]. This could be because component phages complement each other, possess different infection mechanisms, and may recognize different receptors [40]. The three phage components in AB-SA01 were shown to broaden the spectrum of activity and complement each other [24].

The topical administration of AB-SA01 exhibited an effect similar to that observed with vancomycin in hastening infected wound healing. The decrease in wound size also correlated well with the decrease in bacterial load in each of the treatment groups, confirming that bacterial counts differences measured in swab samples reflected the bacteria load in vivo. Wound size measurement showed a significant difference (p < 0.001) between the treatment and control groups. More than 85% of uninfected phage-treated, infected phage-treated, and infected vancomycin-treated mice wounds combined were completely closed, and hair re-growth was observed on some of the wounds. No significant difference (p > 0.05) was observed in wound closure between the three treatment groups.

AB-SA01 was well tolerated by the mice, as shown by the lack of clinical abnormalities such as changes in the well-being of mice or evidence of anaphylactic reactions due to harmful response to phages. This finding is in line with reports on the safety of phage therapy [35, 36, 41–43]. Adverse effects due to rapid bacterial lysis such as bacterial rebound and circulatory shock due to the release of large quantities of toxins when considerable numbers of bacteria are lysed [42], or anti-inflammatory responses [44, 45] were not observed. The absence of adverse effect was expected as the AB-SA01 products used in this experiment is a well-characterized phage cocktail, produced under cGMP standards, and which has been approved by the U.S. Food and Drug Administration (FDA) and Australia’s Therapeutic Goods Administration (TGA) for use in clinical phase I trials and single-patient emergency treatment [24, 43].

## Conclusion

The treatment of diabetic wound infections caused by MDR *S. aureus* using topical application of phage cocktail AB-SA01 is effective, as shown by bacterial load reduction and wound closure. The findings of this study show that phages represent a potentially effective topical treatment for diabetic ulcers infected with antibiotic-resistant pathogens.

## Methods

### Bacterial culture

MDR *S. aureus* isolate 63–2498 (MDR SA63–2498) collected from an Adelaide DFU patient, was identified using matrix-assisted laser desorption/ionization time-of-flight mass spectrometry (MALDI TOF MS) (Bruker Daltonics Biotyper, Bruker Pty. Ltd., Victoria, Australia). MDR SA63–2498 was tested using a VITEK® 2 system (bioMérieux, New South Wales, Australia) and demonstrated resistance to multiple antibiotics, including benzylpenicillin, oxacillin, ciprofloxacin, erythromycin, and clindamycin and susceptibility to vancomycin. MDR SA63–2498 was susceptible to lysis by AB-SA01 and each of its component phages in planktonic and biofilm states during the pilot study (data not shown). A single colony of MDR SA63–2498 grown on a mannitol salt agar (MSA) (Thermo Fisher Scientific, South Australia, Australia) plate was taken and grown in 3 ml trypticase soy broth (TSB) (Thermo Fisher Scientific, South Australia, Australia) overnight with shaking at 160 rpm and 37 °C. One milliliter from the TSB overnight broth culture was adjusted to an optical density (OD) of 0.7 at OD_600_ using a SP-830+ Metertech spectrophotometer (Adelab, South Australia, Australia) that corresponds to 2.3 log_10_ (CFU/ml) through dilution with sterile physiological saline. The adjusted broth culture was centrifuged at 3000 rpm, and pelleted cells were washed twice and resuspended with 1 ml saline. This suspension was used for inoculation into mice’s wounds.

### Phage cocktail

Phage cocktail AB-SA01 was provided by AmpliPhi Biosciences Corporation (now Armata Pharmaceuticals, Inc.). It is a combination of three *Myoviridae* phages designated J-Sa36, Sa83, and Sa87. The mean titer was 9.3 log_10_ (PFU/ml) for J-Sa36 and Sa83, 9.0 log_10_ (PFU/ml) for Sa87, and 9.1 log_10_ (PFU/ml) for AB-SA01 on *S. aureus* laboratory strains RN4220 and SA6538 using plaque assay [24].

### Laboratory animal management

Female Balb/c mice were obtained from the Animal Resources Centre, Perth, Western Australia. All experiments were approved by the Animal Welfare Committee, Flinders University/Southern Adelaide Local Health Network, and carried out in compliance with the ARRIVE guidelines [46]. Mice were kept at the College of Medicine and Public Health Animal Facility, Flinders University, at 22 ± 3 °C and 55 ± 5% humidity under a 12:12 h light-dark cycle in Tecniplast GM500 Mouse IVC Greenline cages (Tecniplast Australia Pty Ltd., New South Wales, Australia) using corncob bedding. Mice were kept in specific pathogen-free conditions. Mice were provided water and meat-free rodent maintenance diet (Glen Forrest Stockfeeders, Western Australia, Australia) ad libitum. The research was conducted consistent with the Australian Code for the Care and Use of Animals for Scientific Purposes, 8th edition, 2013.

Mice were weighed 2–3 times each week and monitored for hunching, ruffled coat, lethargy, cold to touch, crinkling of skin, sunken eyes, and rapid or labored breathing at least once daily. Mice that showed over 15% weight loss or were critically ill were euthanized. Vancomycin (Sigma-Aldrich Corporation, New South Wales, Australia) was assessed for possible toxicity on six mice in a separate pilot study at 150 mg/kg dose rate, twice daily IP, for five consecutive days as described [47]; these mice did not display any apparent adverse reaction. At the end of the experiment, mice were euthanized using 3% isoflurane, followed by cervical dislocation. Post-mortem examination of the external surfaces and visceral organs was conducted following an established procedure [48].

### Induction of diabetes in mice

The diabetes mouse model was used to mimic the human diabetes setting, the source of the experimental bacteria isolates. A total of 48 female 8-week-old Balb/c mice, housed in groups of 5, received streptozotocin (STZ) (Sigma-Aldrich Corporation, New South Wales, Australia) following an established protocol [26]. Balb/c mice are among the least susceptible to STZ toxicity because of their relatively high pancreatic β-cell mass due to their large number of islets [49]. Female mice are relatively resistant to the glucotoxicity of STZ compared to males because sex steroids protect them from β-cells injury [50]. Besides, STZ-diabetic nephropathy is more pronounced in male mice compared to females [49]. STZ is a naturally occurring alkylating antineoplastic agent that is toxic to pancreatic insulin-producing cells. It is used to treat pancreas islet cell carcinoma and to induce diabetes mellitus in laboratory rodents [25].

After 4 h of fasting, each mouse received an IP injection of 50 mg/kg freshly dissolved STZ in 0.05 M citrate buffer pH 4.5 once daily for five consecutive days. Non-fasting blood glucose levels (BGL) were checked every 2–3 days by tail vein bleeding after applying 3% lidocaine/prilocaine anesthetic cream. BGLs were measured using an Accu-Chek Performa (Roche Diabetes Care Australia, New South Wales, Australia) blood glucose meter. Mice with non-fasting BGL < 13.9, between 13.9 and 22.2, and ≥ 22.2 mmol/L on at least two different days were categorized as normal glycaemic, moderate hyperglycaemic, or severe hyperglycaemic status, respectively, as described earlier [51]. Mice were treated with 1.0 IU NovoMix® 30 (Novo Nordisk, New South Wales, Australia) insulin subcutaneously daily, from the second week of diabetes manifestation, to ameliorate the hyperglycaemic effects of diabetes and maintain body weight [52].

### Excisional wound infliction

Diabetic mice were provided with lemon-flavored paracetamol in drinking water at 1.34 mg/ml 3 days prior to wound infliction and for the entire experimental period. An established rodent wound infection model [29] was used. Mice were anaesthetized using 3% isoflurane inhalation and maintained on 1.5% during surgery. Hair was removed on the dorsal skin using electric clippers and depilatory cream, and skin sterilized using 70% ethanol. Mice were given a single 0.05 mg/kg buprenorphine injection subcutaneously before the incisions. Hydrating eye drops were applied during anesthesia.

A skin wound extending through the *Panniculus carnosus* muscle was inflicted using a 6 mm sterile biopsy punch and fine scissors. The bilateral wounds were at 10 mm either side of the midline and 30 mm from the base of the skull. Swab samples were collected from each wound to assess for existing *S. aureus.* A sterile 1 mm thick silicone splint with a 7 mm diameter circular hole at the center was applied over the wound and sutured onto the skin concentric with the wound using 5–0 nylon suture. Cyanoacrylate glue was used to fix the silicone splint to the skin before suturing. The silicon splints were removed at the end of the experiment.

### Wound infection and treatment

Mice were randomly assigned into *S. aureus*-inoculated phage-treated (*n* = 8), *S. aureus*-inoculated vancomycin-treated (*n* = 6), *S. aureus*-inoculated saline-treated (*n* = 7), and saline-inoculated phage-treated (*n* = 8) groups. *S. aureus*-inoculated mice were infected with 50 μl suspension containing 6.7 log_10_ (CFU) of MDR SA63–2498 prepared as above. Mice in the saline-inoculated group received 50 μl of saline. Suspensions were applied directly into each wound, overlaid with gauze, and covered with Opsite sterile transparent wound dressing, which adhered to the silicon splint and retained in place the gauze dressing. Post-infection, each mouse was kept in a single housing system. On days 3, 5, and 7 post-infection, the Opsite was removed, and swab samples were taken. Swab samples were taken by scrubbing the surface of each wound by rotating three times clockwise with enough pressure to produce a small amount of exudate and inserted into a separate tube of 1 ml TSB. The tube was vortexed (with the swab inside), to distribute the bacteria in the TSB solution evenly, for 5 s, and a 100 μl aliquot was used for 10-fold serial dilutions. Swab samples were kept at + 4 °C and processed for the bacterial count within 4 h of collection.

Treatments were administered after sample collection commencing on day 3. Gauze patches (10 × 10 mm) soaked with 70 μl AB-SA01, equivalent to 7.9 log_10_ PFU, or 70 μl saline solutions for control and vancomycin-treated mice, were applied to wounds and covered with Opsite, on days 3, 5, and 7 post-infection. Vancomycin-treated mice received 150 mg/kg vancomycin IP twice daily for five consecutive days, as described [47]. Topical vancomycin was not an option because its best topical dose is unknown, it has poor tissue penetration, and could contribute to vancomycin resistance [30, 53]. Both wounds of a mouse received the same infection and treatment.

### Assessment of treatment effect on bacterial load and wound healing

Wound size was measured in duplicate from multiple directions using a digital Vernier caliper by tracing the leading edge of epithelium within the wound [54]. The wound size on the excision day was defined as the original wound size. One hundred microliters of 10-fold serially diluted swab sample suspensions were mixed with 3 ml trypticase soy soft agar containing 0.65% bacteriological agar for even distribution of the bacteria [55] and cultured on MSA to assess the bacterial load. After 24 h of incubation at 37 °C aerobically, colony count was performed on plates with 30–300 colonies as recommended [56]. The bacterial population was calculated using the formula B = N/d where B = number of bacteria, N = average number of colonies, and d = dilution factor.

### Data management and statistical analysis

Data were double entered, encoded, and stored using Microsoft Excel Spreadsheet. STATA (version 16) software was used for statistical analysis. Data are reported in terms of mean ± standard error. CFU data are expressed as logarithm-transformed values (log_10_ (CFU /ml)) over time. A comparison of experimental groups was performed using a one-way analysis of variance (two-tailed) or paired ‘t*-test*’. A *p* < 0.05 value was considered statistically significant.

## Data Availability

The datasets used and/or analyzed during the current study are available from the corresponding author on reasonable request.

## Abbreviations

BGL: Blood glucose level
CFU: Colony-forming units
cGMP: Current good manufacturing practice
DFU: Diabetic foot ulcer
FDA: Food and Drug Administration (FDA)
IP: Intraperitoneal
MALDI TOF MS: Matrix-assisted laser desorption/ionization time-of-flight mass spectrometry
MDR: Multidrug-resistant
MRSA: Methicillin-resistant *S. aureus*
MSA: Mannitol salt agar
OD: Optical density
PFU: Plaque forming unit
RPM: Revolutions per minute
*S. aureus*: *Staphylococcus aureus*
STZ: Streptozotocin
TGA: Therapeutic Goods Administration
TSB: Trypticase soy broth (TSB)
US: United States
